# In Situ Assessment of Uplink Duty Cycles for 4G and 5G Wireless Communications

**DOI:** 10.3390/s24103012

**Published:** 2024-05-09

**Authors:** Günter Vermeeren, Leen Verloock, Sam Aerts, Luc Martens, Wout Joseph

**Affiliations:** Department of Information Technology, Ghent University/imec, 9052 Ghent, Belgium; leen.verloock@ugent.be (L.V.); luc1.martens@ugent.be (L.M.)

**Keywords:** 5G NR, EMF exposure, duty cycle, uplink, smartphone

## Abstract

In this presented study, we measured in situ the uplink duty cycles of a smartphone for 5G NR and 4G LTE for a total of six use cases covering voice, video, and data applications. The duty cycles were assessed at ten positions near a 4G and 5G base-station site in Belgium. For Twitch, VoLTE, and WhatsApp, the duty cycles ranged between 4% and 22% in time, both for 4G and 5G. For 5G NR, these duty cycles resulted in a higher UL-allotted time due to time division duplexing at the 3.7 GHz frequency band. Ping showed median duty cycles of 2% for 5G NR and 50% for 4G LTE. FTP upload and iPerf resulted in duty cycles close to 100%.

## 1. Introduction

With fifth-generation (5G) wireless communication technology, new and higher frequency bands, beamforming, and massive multiple-input multiple-output (MIMO) techniques are introduced to further enhance the efficiency of the radio access network (RAN), allowing ultra-reliable communications, broadband communication, and massive deployments of wireless IoT devices. As electromagnetic field (EMF) exposure quantities are time-averaged quantities [1,2], knowledge of the duty cycle—a measure of the fraction of time during which a mobile phone is transmitting—for realistic use cases plays a key role in the accurate evaluation of EMF exposure assessments.

In this study, we focused on assessing the uplink (UL)—from a smartphone towards a radio access network—duty cycle of a 5G-enabled smartphone. Before a smartphone is introduced to the market, the smartphone must comply with exposure limits in terms of the local specific absorption rate (SAR) averaged over 10 g [1] or 1 g of body tissue [2] following standardized measurement methods [3]. However, in realistic use cases, a smartphone will almost never be continuously transmitting, i.e., at a duty cycle of 100%, depending on the application or service used. The assessment of realistic EMF exposure to the uplink of a smartphone thus requires knowledge of the duty cycle. Time-averaged output powers of user devices have been investigated for GSM [4], 3G [5,6], 4G [7], and 5G [8] devices. Furthermore, Gati et al. [9] investigated the duality between UL local and DL whole-body exposure, and Joseph et al. [10] determined the duty cycle for Wireless Local-Area Networks (WLANs) for several data and voice applications and in a variety of environments. Such knowledge of realistic duty cycles will aid the determination of upper bounds on the time-averaged output powers of mobile equipment for different applications. The novelties of this study are as follows:

1. A method for assessing uplink duty cycles using the drive test tool QualiPoc (Rohde & Schwarz, Munich, Germany);
2. An assessment of the in situ UL duty cycle of a smartphone for 4G and 5G New Radio (5G NR) for typical voice and data applications in the proximity of a 4G/5G base-station site in Belgium;
3. An investigation of the influence of the Physical Uplink Shared Channel (PUSCH) transmit power, the reference signal received power (RSRP), and the Modulation Coding Scheme (MCS) on the uplink duty cycle.

Section 2, the Materials and Methods section, presents the method for assessing duty cycles in realistic usage patterns for voice, video, and data applications based on the parameters obtained from the drive test tool. Section 3 discusses the observed duty cycles as well as uplink parameters that provide more profound insights in the assessment of duty cycles. Finally, Section 4 puts this study into a broader context and points out the limitations of this study. Section 5 concludes this in situ study and proposes the next steps beyond the scope of the present study.

## 2. Materials and Methods

**Figure 1:**
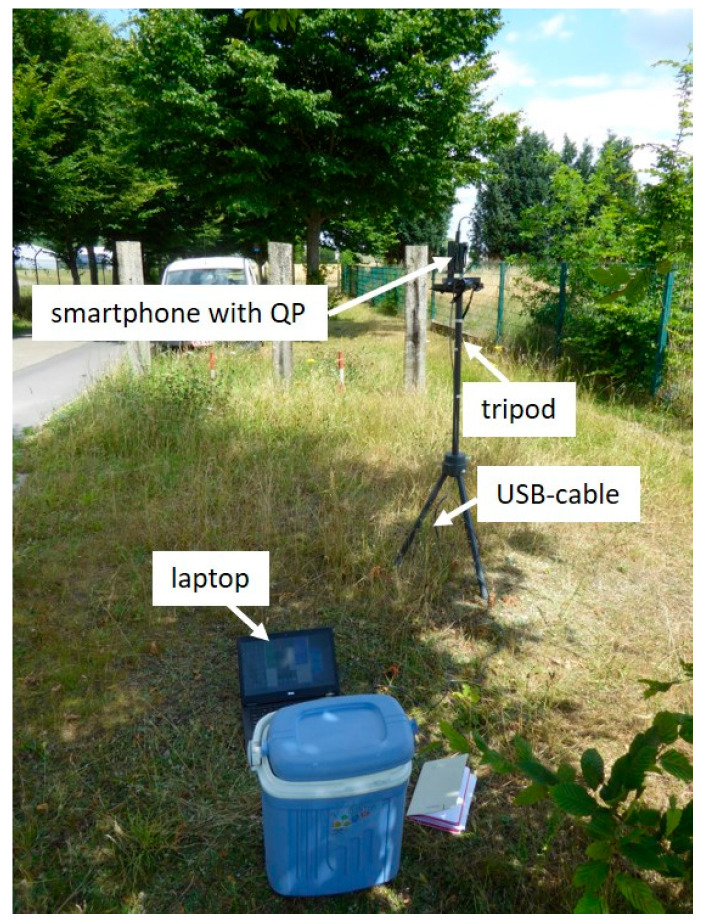
Measurement setup consisting of a smartphone with QualiPoc on a plastic tripod. The smartphone was connected via a USB cable to the laptop for remote control with the Vysor app.

The uplink duty cycle of a smartphone under realistic exposure conditions was measured using the setup shown in Figure 1. This setup consisted of a smartphone with the drive test tool QualiPoc (Rohde & Schwarz, Munich, Germany), a tripod, and a laptop that was connected to the smartphone using a USB cable. To avoid interference from the operator, the smartphone was connected to the laptop with a 5 m USB cable, allowing the operator to remotely control it using the Vysor app. The drive test tool QualiPoc logged the relevant parameters of the smartphone chipset to determine the uplink duty cycle. We measured the duty cycle for different smartphone applications at ten positions using a 4G/5G base-station antenna. During the measurements, the smartphone was placed in a plastic holder on a tripod at a height of 1.5 m (Figure 1). 

**Figure 2:**
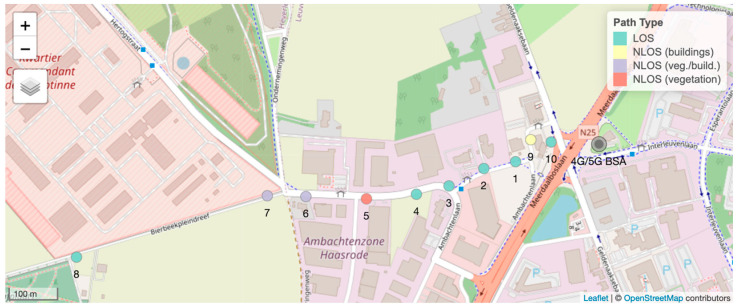
The measurement positions (colored dots). The positions were in Line-of-Sight (LOS) or Non-Line-of-Sight (NLOS) paths in relation to the base-station antenna (black circle). In the NLOS path, there was no 5G connection.

This in situ measurement campaign was performed in the vicinity of a collocated 4G LTE and 5G NR base station in Leuven, Belgium (Figure 2). Measurements were conducted at ten positions covering Line-of-Sight (LOS) and Non-Line-of-Sight (NLOS) paths between the smartphone and the base-station antenna (Figure 2). The distance between the base-station antenna and the measurement positions together with the corresponding path types are listed in Table 1.

At each measurement position, six use cases (UCs) were assessed for 4G LTE and five UCs were assessed for 5G NR. The use cases together with brief descriptions of each case are listed in Table 2. The voice call in UC1 was only performed for LTE [Voice over LTE (VoLTE)] because Voice over NR (VoNR) was not available. In total, 110 experiments were performed. 5G NR used the time division duplexing (TDD) band n78 at 3.7 GHz. 4G LTE used the frequency division duplexing bands n3, n7, and n20 at 1800 MHz, 2100 MHz, and 800 MHz.

We used a Samsung S20+ 5G (SM-G986) smartphone with the drive test tool QualiPoc (Android Ver. 21.03 SP2, Rohde & Schwarz, Germany). The smartphone only allowed us to lock onto 4G LTE technology and not onto 5G NR since the 5G NR network was non-standalone (NSA).

Table 3 shows a non-exhaustive list of parameters reported by QualiPoc Android Ver. 21.03 SP 2 [11] relevant for this study.

The interval during which results are reported in QualiPoc, also called the observation period, depends on the chipset of the used phone and on the technology used. The phone used during our in situ measurements was a Samsung S20 with an Exynos chipset. For 4G, values were randomly picked from those reported (from the chipset) in the observation period and then averaged. Thus, for 4G, the observation period was random and depended on the parameters considered: for the uplink power (i.e., PUSCH Tx power), the averaged observation period was 200 ms, whereas this was 400 ms for the transport block size (i.e., PUSCH TBS). For 5G, the observation period was 500 ms.

As there was no direct parameter reported relating to the number of transport blocks (TBs) for 5G NR (see Table 3), we opted to use an indirect way to calculate this parameter for both 4G LTE and 5G NR. Since the throughput is equal to the number of TBs times the size of those transport blocks, and both the throughput and transport block size (TBS) were recorded using QualiPoc (Table 4), we used the following equation:

Average number of TBs [/s] = Throughput [MBit/s] × 10^6^ [bit/Mbit]/TBS [bit]. (1)

Moreover, as the number of TBs per transmission time interval (TTI) is either 0 or 1 (in the case of no spatial multiplexing)—in other words, there is either a transmission or no transmission—the ratio of the average number of TBs and the number of TTIs per observation period can be considered the duty cycle (DC) (i.e., the ratio of TB transmissions to elapsed TTIs):

DC [-] = Average number of TBs [/s]/(# TTI/s),(2)

with the number of TTIs per second (# TTI/s).

For 4G, the TTI is fixed at 1 ms, resulting in a maximum number of 1000 TTIs per second. For 5G, the TTI depends on the SCS (sub-carrier spacing) of the 5G signal, with values between 1 ms (for an SCS of 15 kHz) and 62.5 µs (for an SCS of 240 kHz) corresponding to a maximum number of TBs per second between, respectively, 1000 and 16,000. Based on the configuration of the SCS, which was 30 kHz during our in situ tests, a TTI of 500 ms was expected. In [11], only information about the TTI for 4G was mentioned, but no information was available about the TTI considered by QualiPoc.

To evaluate the TTI used by QualiPoc, a QualiPoc test during a continuous FTP upload of a huge file was performed with a 4G and a 5G connection. During these tests, a continuous UL stream of TBs at maximum capacity, i.e., one TB for each TTI, was expected to result in the maximum possible number of TBs per second.

**Figure 3:**
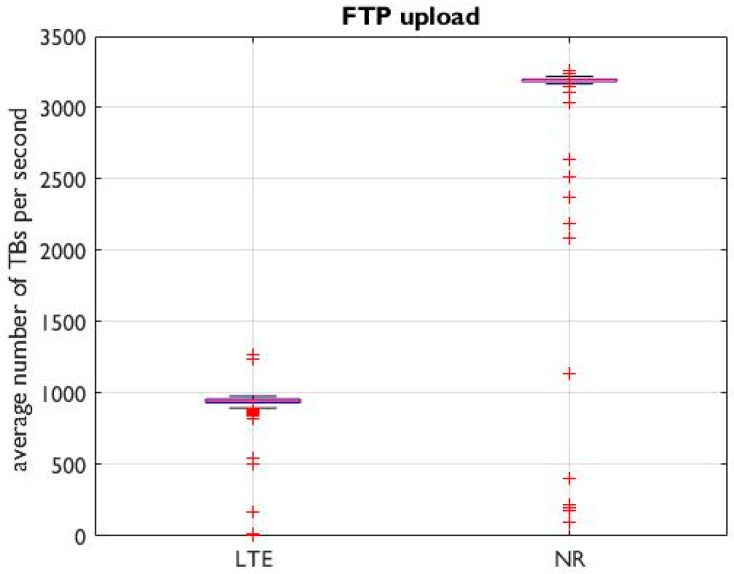
The average number of TBs per second (Equation (1)) at position 2 during our 2-min QP campaign involving a continuous FTP upload of a huge file (4G LTE and 5G NR).

Figure 3 shows a boxplot of the average number of TBs per second for the continuous FTP upload of a huge file, calculated using Equation (1). For LTE, we observed values up to 1000 (with a few outliers, probably due to misreporting), as expected (TTI = 1 ms). For 5G, we observed values up to 3200 (99th percentile). This did not correspond to the maximum expected value of 400 (=2000/5) for a 5G configuration with an SCS of 30 kHz (TTI = 0.5 ms) and a TDD factor of 5 (DDDSU slot configuration, i.e., 1/5 UL and about 4/5 DL). Consequently, this showed that the TTI for 5G used by QualiPoc is fixed at a duration of 62.5 µs, resulting in a maximum number of TBs per second of 3200 (=16,000/5). Consequently, in the subsequent analyses, a TTI of 62.5 µs was considered for 5G signals.

## 3. Results

In this section, we discuss the UL duty cycles calculated from the parameters reported from the drive test tool QualiPoc, as well as other relevant parameters that offer enhanced insights for evaluating exposure: the use of bandwidth, the PUSCH transmit power, the transport block size, the number of bits per resource block (RB), and the reference signal received power (RSRP). We conclude this section with a summary of the duty cycles per application and communication technology. 

### 3.1. Duty Cycles

**Figure 4:**
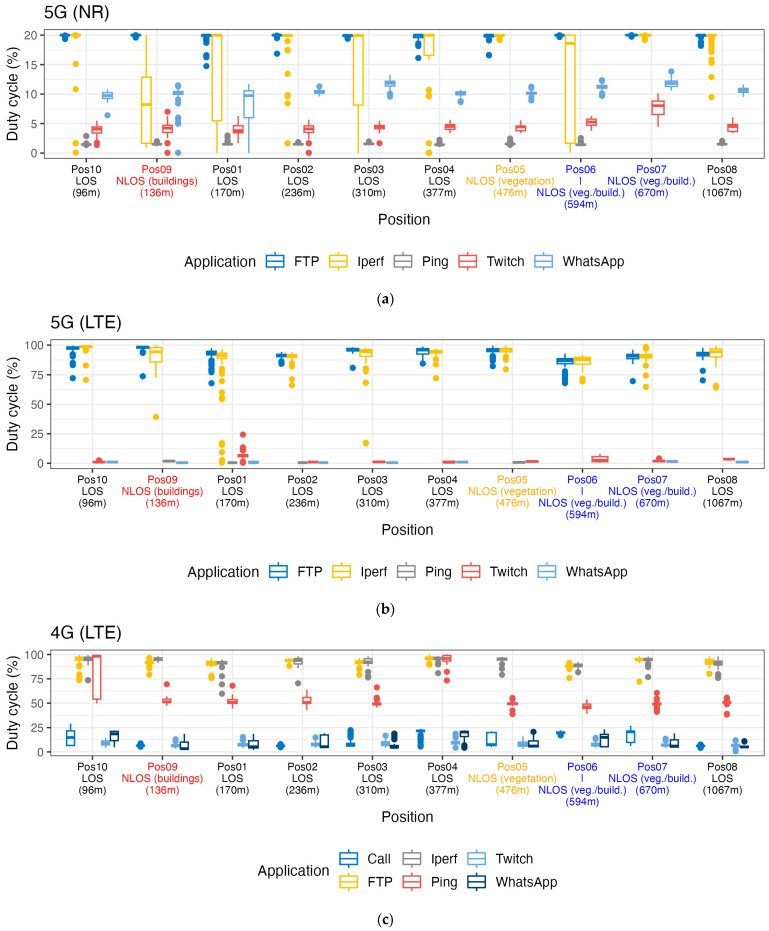
One-second-averaged duty cycles as a function of distance to the base-station antenna per use case and per technology: (**a**) 5G-NR, (**b**) 5G-LTE, and (**c**) 4G-LTE. Remark that for 5G NR, there was an upper duty cycle limit of 20%, considering the used TDD slot format DDDSU. The color of the labels on the horizontal axis indicates the path type, i.e., red, orange, and blue indicates NLOS (buildings), NLOS (vegetation), and NLOS (veg./build.), respectively.

We calculated the duty cycles according to Equation (2). Figure 4 shows the boxplots of the calculated duty cycles for 5G NR, 5G LTE (to distinguish it from LTE in the case of 4G connectivity, we define 5G LTE as LTE in the case of the 5G NSA), and 4G LTE at the investigated positions. The measurement positions were ordered as a function of their horizontal distance from the base-station antenna (see Table 1, where for each position, we mention the distance and the type of path). We observed that data-intensive applications, as expected, resulted in the largest duty cycles: for 5G-NR, the FTP upload showed a duty cycle of 19.99% for all measurement positions and iPerf also showed duty cycles larger than 19.9% for all positions, except at Pos 6 (18.6%) and Pos 9 (8.2%). The duty cycles for Ping and Twitch were below 1.6% and 8%, respectively. For WhatsApp, we observed duty cycles between 9.7% (Pos 10) and 11.9% (Pos 7). For 5G-LTE, the uplink duty cycle for FTP and iPerf exceeded 90%, except at Pos 6 (FTP upload: 86.9%; iPerf: 87.7%); the duty cycle for Ping and WhatsApp was below 2% for all positions; and for Twitch, the duty cycle ranged between 0.8% (Pos 1) and 1.9% (Pos 7). We note that the maximum duty cycle for 5G NR was 20%, based on the used TDD slot format DDDSU. For 4G-LTE, the uplink duty cycle for FTP and iPerf exceeded 90%, except at Pos 6 (FTP upload: 87.9%; iPerf: 88.8%); Ping showed duty cycles around 50%, except at Pos 4 and Pos 10, where a duty cycle above 95% was observed; Twitch showed duty cycles below 10%; the duty cycles for the WhatsApp (VoIP) and VoLTE calls followed a similar trend, with duty cycles below 10% except at Pos 4 (WhatsApp: 20.4%; Call: 21.6%), Pos 6 (WhatsApp: 15.0%; Call: 19.7%), and Pos 10 (WhatsApp: 18.6%; Call: 14.8%). 

Based on this limited set of ten measurement positions, no trend in the uplink duty cycles could be determined as a function of distance to the base-station antenna. Comparing the uplink duty cycles for the different path types, only Pos 7 indicated an increase in the uplink duty cycle for Twitch of about 77% (comparing Pos 7 with Pos 8) for 5G-NR. The relation between the DCs and other parameters will be discussed in the next section.

### 3.2. Use of Bandwidth

**Figure 5:**
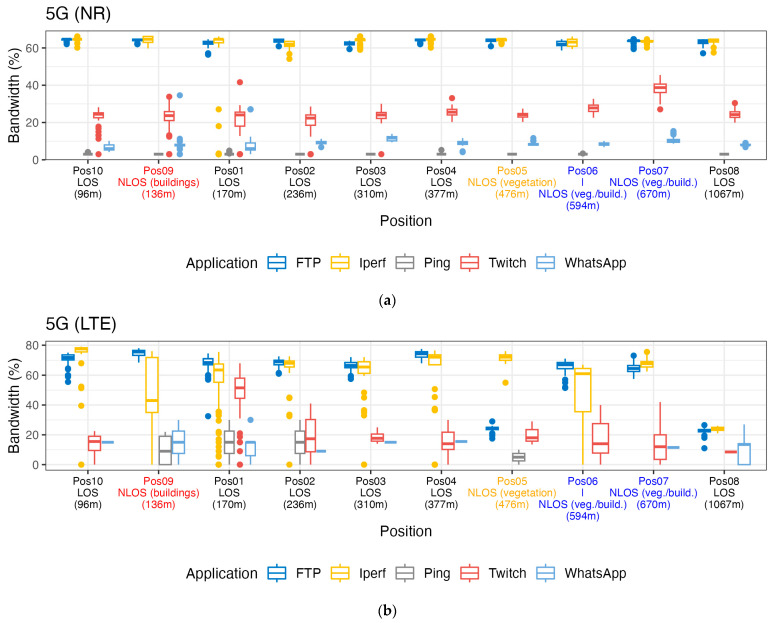

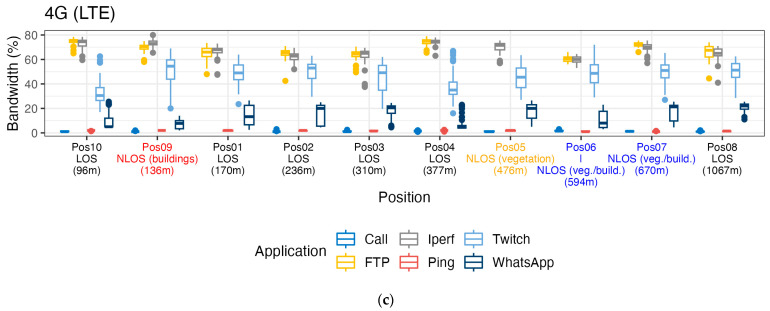
One-second-averaged uplink bandwidth fractions as a function of distance to the base-station antenna per use case and per technology: (**a**) 5G-NR, (**b**) 5G-LTE, and (**c**) 4G-LTE. The color of the labels on the horizontal axis indicates the path type, i.e., red, orange, and blue indicates NLOS (buildings), NLOS (vegetation), and NLOS (veg./build.), respectively.

For each experiment, we averaged the number of UL resource blocks normalized to the bandwidth (BW). We assumed bandwidths of 10 MHz, 20 MHz, and 50 MHz for 4G LTE, 5G LTE, and 5G NR, respectively. In general, we observe in Figure 5 that data-intensive applications, i.e., FTP upload and iPerf, consume a larger bandwidth than low-data applications, i.e., Ping and WhatsApp, and that 5G NR uses fewer frequency resources than LTE:

- WhatsApp: median value of 8.7% for 5G NR versus 11% for LTE;
- Twitch: median value of 24.4% for 5G NR versus 49% for LTE;
- FTP: median value of 63.5% for 5G NR versus 68% for LTE;
- iPerf: median value of 63.9% for 5G NR versus 68% for LTE.

5G NR only consumed slightly larger bandwidth resources than LTE for Ping, with median values of 2% and 3% for LTE and 5G NR, respectively. The 4G call or VoLTE used a very small fraction of the bandwidth (median value of 1%) compared to the WhatsApp call (11% for LTE and 8.7% for 5G NR).

We did not observe a trend in the bandwidth usage as a function of distance to the base-station antenna. 

### 3.3. PUSCH Transmit Power

**Figure 6:**
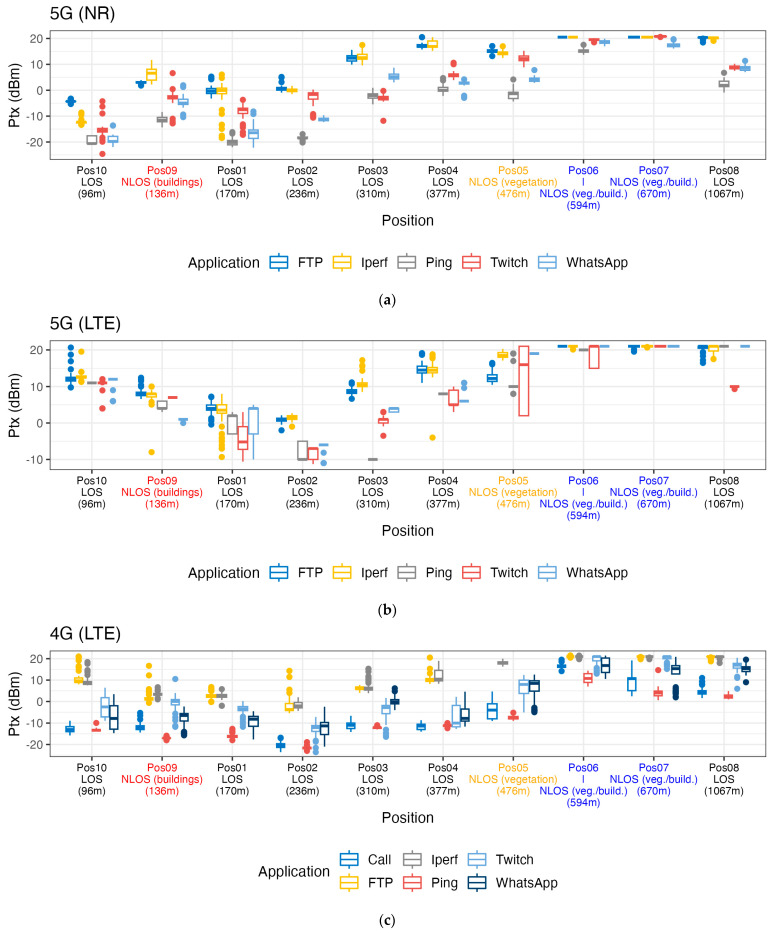
Boxplots of the one-second median PUSCH transmit powers as a function of distance to the base-station antenna per use case and per technology: (**a**) 5G-NR, (**b**) 5G-LTE, and (**c**) 4G-LTE.

A key parameter in EMF exposure is the transmit power of the device. Figure 6 shows the boxplots of the PUSCH transmit power (P_tx_) in dBm as a function of distance to the base-station antenna. Again, the path type between the smartphone and base-station antenna is mentioned in this figure. For each of the experiments, we plotted the median PUSCH Tx power (i.e., the reported transmit power independent of the use case, indicating a set value, not a time average), as shown in Figure 6. In order of magnitude, we observed similar PUSCH Tx powers for 5G NR and 4G LTE, ranging between −20 dBm and 20 dBm. As for the duty cycle and bandwidth, we observe that data-intensive applications show the largest values for the PUSCH transmit power. For 5G NR, we also observe that the transmit power increased with the distance of the smartphone to the base-station antenna. When communicating over LTE, irrespective of the network mode (4G or 5G), the PUSCH transmit power decreased up to Pos 2 (236 m from the base station) and increased with the distance for positions further away. Looking at the influence of the path type on the transmit power, there is an indication of a possible increase in transmit power for 5G NR at Pos 9 (NLOS due to buildings; see Figure 6a) and for 5G and 4G LTE at Pos 6 (NLOS due to buildings and vegetation).

### 3.4. Impact of the Modulation Coding Scheme

**Figure 7:**
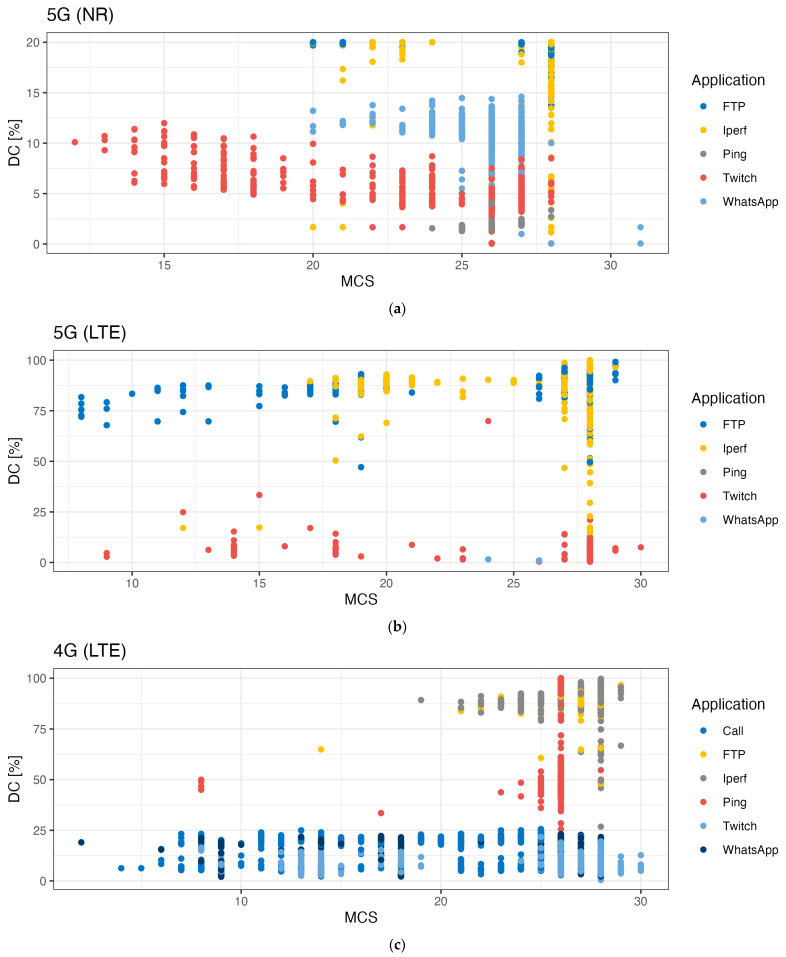
Impact of the MCS per use case on the DC for (**a**) 5G NR, (**b**) 5G LTE, and (**c**) 4G LTE. Remark that the MCS value depends on the technology and modulation used.

**Figure 8:**
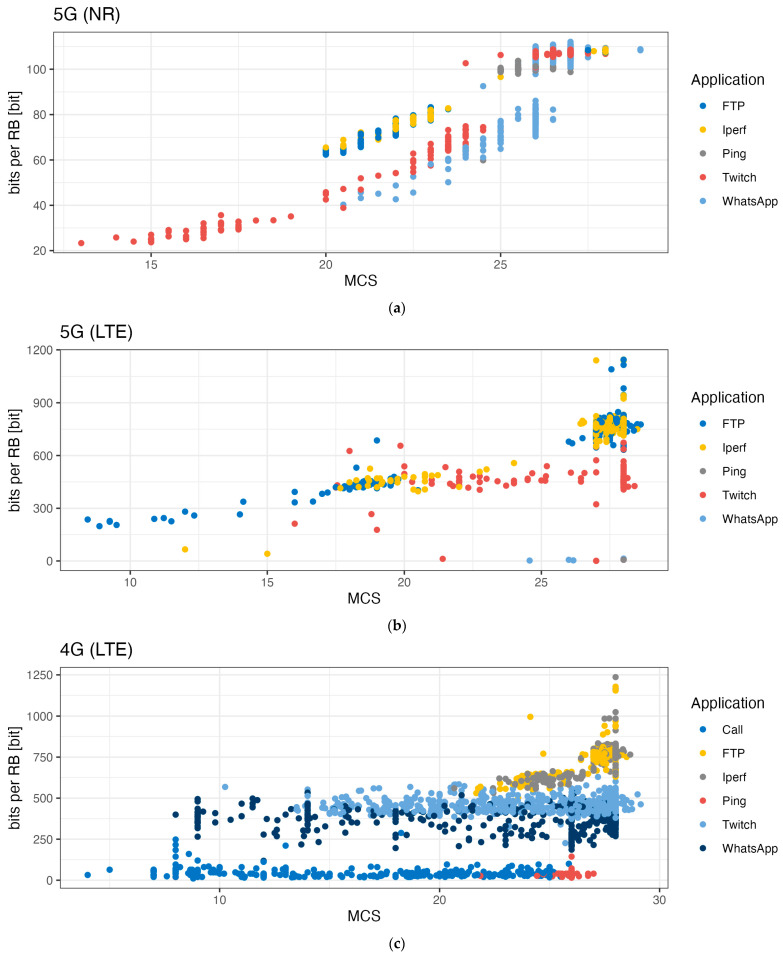
Impact of the MCS per use case on the bits per RB for (**a**) 5G NR, (**b**) 5G LTE, and (**c**) 4G LTE. Remark that the MCS value depends on the technology and modulation used.

**Figure 9:**
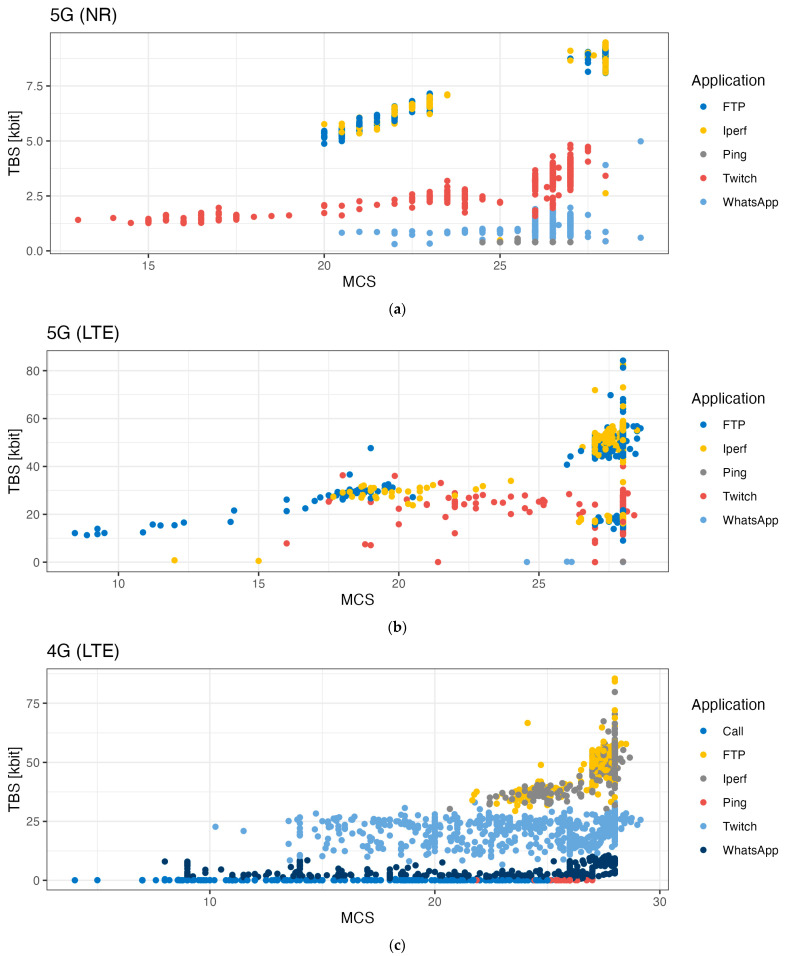
Impact of the MCS per use case on the bits per TBS for (**a**) 5G NR, (**b**) 5G LTE, and (**c**) 4G LTE. Remark that the MCS value depends on the technology and modulation used.

Figure 7, Figure 8 and Figure 9 show the influence of the Modulation Coding Scheme (MCS) on the duty cycles, TBS, and the number of bits per RB for 5G NR, 5G LTE, and 4G LTE. 

For 5G NR, the MCS value did not seem to impact the duty cycle except for the iPerf and Twitch (video upload stream) use cases (see Figure 7a). In the case of iPerf, we observed an increase in the spread of the calculated duty cycle data with increasing MCS values (min–max spread of 1% to 99% for MCS values from 20 to 28). For Twitch, the duty cycle decreased with increasing MCS values (median TBS size from 1534 bits to 4963 bits for MCS values from 11 to 28). Figure 8a shows that an increase in the MCS value resulted in an increase in the number of bits per resource block. Figure 9a shows the TBS as a function of the MCS: increasing MCS values resulted in an increase in TBS. With higher MCS values, a larger amount of data can be transmitted at the same time. 

For 4G LTE, the duty cycles also do not show a trend when assessed as a function of the MCS value (see Figure 7). 

### 3.5. Impact of RSRP on the PUSCH Transmit Power per RB and Duty Cycle

**Figure 10:**
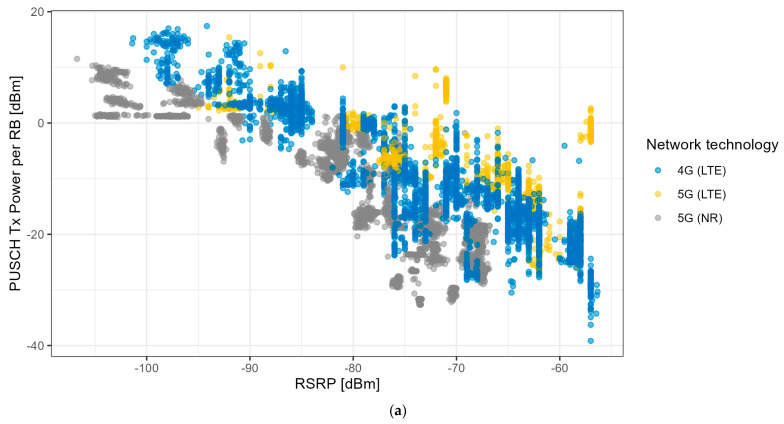

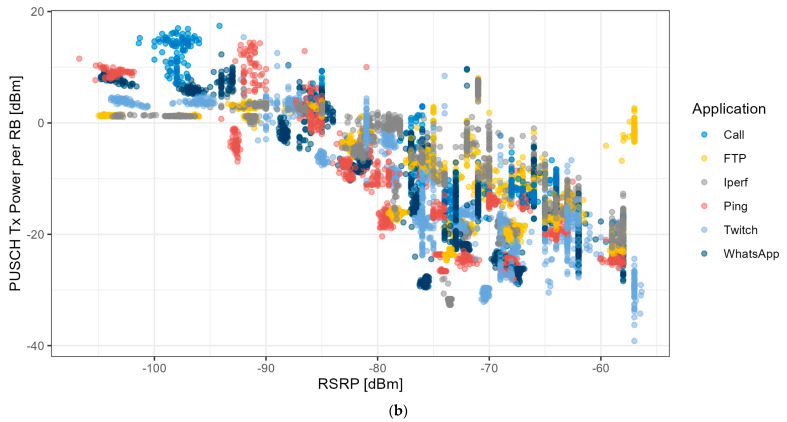
Impact of the RSRP on the PUSCH transmit power per RB for (**a**) the network technologies and (**b**) the applications.

Figure 10 shows the PUSCH Tx power per RB as a function of the RSRP. We can clearly observe that the PUSCH Tx power per RB decreases with increasing RSRP values irrespective of the technology used, i.e., 4G LTE or 5G NR, or the use case (application). We observe that the 5G NR PUSCH Tx power per RB was lower than that for 4G LTE (see Figure 10a). We remark that the number of bits per RB was also lower for 5G NR compared with 4G LTE. We also observe large variations in the PUSCH Tx power per RB for constant RSRP values (up to 20–30 dB), and vice versa. Figure 10b shows the PUSCH Tx power per RB as a function of the RSRP for each of the considered use cases: the PUSCH Tx power per RB decreases with increasing RSRP values, except for when the maximum transmit power is reached (for RSRP values between −95 and −105 dBm and for the FTP and iPerf use cases).

### 3.6. Summary of Uplink Duty Cycles in 5G NR, 5G LTE, and 4G LTE

Table 4 lists the duty cycles per application (or use case) and technology calculated from the in situ measurements recorded at Leuven, Belgium. The duty cycles for Twitch, VoLTE, and WhatsApp ranged between 4% and 22% in time. We remark that, due to the TDD in 5G NR, 5G NR used a higher fraction of the UL-allocated slots. Ping had median duty cycles of 4% and 50% for 5G NR and 4G, respectively. We remark that for 4G, the 95th percentile of the duty cycle equaled 99%. Finally, the FTP upload and iPerf applications nearly fully used the UL-allocated time, as their median duty cycles equaled close to 92–93% for 4G and 20% for 5G NR. The mean duty cycle can be regarded as an estimate of the time-averaged duty cycle for the considered application per network technology.

## 4. Discussion

In this study, we measured in situ the uplink duty cycles of a smartphone for 5G NR and 4G LTE for a total of six use cases covering voice, video, and data applications. The duty cycles were measured at ten positions near a 5G base-station site in Belgium. The distance of the measurement positions to the base-station antenna site varied between 95 m and 1067 m. The propagation paths between the smartphone and base-station antenna site were visually identified and covered LOS and NLOS paths. The number of measurement positions were too limited to evaluate the duty cycle as a function of the path type. Nevertheless, some possible trends were observed. 

We remark that we calculated the duty cycles from QualiPoc measurements on a Samsung S20+ with an Exynos chipset. Although we measured the uplink parameters using a specific smartphone, we expect that similar observations would be found with other smartphones with similar operations in terms of transmit power management.

The presented duty cycles were calculated from measurements with a smartphone connected to a live commercial 4G/5G radio access network; these duty cycles can be used to estimate exposure in terms of the specific absorption rate for the considered configurations and similar smartphone operations.

## 5. Conclusions

This study presents the uplink duty cycles and related parameters determined from in situ measurements near a 4G and 5G base-station antenna in Leuven, Belgium. Six use cases were considered for 4G LTE. These use cases were repeated for 5G NR except for the use case ‘Call’, which was VoLTE. 

The duty cycles remained relatively stable during each of the use cases. For Twitch, VoLTE, and WhatsApp, the duty cycles ranged between 4% and 22% in time. For 5G NR, these duty cycles resulted in a higher UL-allotted time due to time division duplexing at the 3.7 GHz frequency band. Ping showed median duty cycles of 2% for 5G NR and 50% for 4G LTE. FTP upload and iPerf resulted in duty cycles close to 100%. 

The 95th percentile of the PUSCH transmit power ranged between 6% and 63% of the maximum value of 23 dBm. We also investigated the impact of the MCS and RSRP on the duty cycle and PUSCH transmit power per resource block: for 5G NR, the duty cycle tended to decrease for increasing MCS values, as expected, whereas for 4G, no clear impact of the MCS on the duty cycle was observed. Finally, the PUSCH transmit power decreased with increasing RSRP values and showed no impact on the duty cycle.

## Figures and Tables

**Table 1:** List of measurement positions with their distance from the base station and the visually perceived path type between the smartphone and the base-station antenna.

Measurement Position	Distance to Base-Station Antenna (m)	Path Type
Pos 1	169.7	LOS
Pos 2	235.8	LOS
Pos 3	310.2	LOS
Pos 4	377.0	LOS
Pos 5	476.4	NLOS (vegetation)
Pos 6	594.1	NLOS (vegetation/buildings)
Pos 7	670.0	NLOS (vegetation/buildings)
Pos 8	1066.9	LOS
Pos 9	136.1	NLOS (buildings)
Pos 10	95.5	LOS

**Table 2:** List of use cases (UCs) and their descriptions.

Use Case	Description
UC1	Voice call (VoLTE) ^1^
UC2	Voice call via WhatsApp (VoIP)
UC3	Twitch video upload stream
UC4	Ping
UC5	Internet Performance Working Group (iPerf) (maximum traffic)
UC6	File Transfer Protocol (FTP) upload of a large file (10 GB)

^1^ Not performed for 5G NR.

**Table 3:** Non-exhaustive list of parameters provided by the Rohde & Schwarz QualiPoc Android Ver. 21.03 SP 2 [11] tool.

Parameter [Unit]	Technology	Name	Description
**Transmit Power [dBm]**	5G NR	PUSCH Tx power	Physical Uplink Shared Channel (PUSCH) transmit (Tx) power of the 5G NR cell.
	4G LTE	(PUSCH) Tx power ^1^	Current (total) transmitting power (on the PUSCH).
	4G LTE	PUCCH Tx power	Current transmitting power on the Physical Uplink Control Channel (PUCCH).
**Number of Transport Blocks [-]**	5G NR	n/a	n/a
	4G LTE	PUSCH number of TBs	Number of transport blocks (TBs) allocated to the UE in a primary cell.
**Throughput [kbps]**	5G NR	Scheduled PUSCH Thp	Resulting PUSCH throughput (Thp) of all transmitted TBs, including non-acknowledged TBs. [12]
	4G LTE	PUSCH throughput	Throughput on the PUSCH of the primary cell, only counting the transport blocks that are sent for the first time.
**Transport Block Size [bit]**	5G NR	Avg PUSCH TBS	Average PUSCH transport block size (TBS) in the observation period.
	4G LTE	PUSCH TBS average	Average transport block size: average number of user data bits transmitted in the TTIs associated with the UE.
**Number of Resource Blocks [-]**	5G NR	PUSCH RB average	Average number of PUSCH RBs used in the observation period.
	4G LTE	Channel quality UL re-source block number ^2^	Uplink resource block (RB) number.

^1^ Parameters ‘Tx power’ and ‘PUSCH Tx power’ were equal for 4G LTE. ^2^ Equal to the parameter ‘4G LTE PUSCH RB average’, but with more samples.

**Table 4:** Summary of the duty cycles: mean, median, and 95th percentile of the considered applications obtained from in situ measurements near a 4G and 5G base-station antenna in Leuven, Belgium. Remark that for 5G NR, there was an upper duty cycle limit of 20%, considering the used TDD slot format DDDSU.

	4G (LTE)			5G (LTE)			5G (NR)		
Application	Mean (DC) (%)	p_50_ (DC) (%)	p_95_ (DC) (%)	Mean (DC) (%)	p_50_ (DC) (%)	p_95_ (DC) (%)	Mean (DC) (%)	p_50_ (DC) (%)	p_95_ (DC) (%)
Call (VoLTE)	12.4	7.2	22.5	-	-	-	-	-	-
FTP	92.5	93.1	97.7	93.0	94.1	98.8	19.9	20.0	20.0
iPerf	93.0	94.1	97.8	91.3	93.3	99.1	17.3	20.0	20.0
Ping	56.4	50.2	98.5	1.0	0.8	2.1	1.5	1.5	1.8
Twitch	8.0	7.2	15.0	5.7	5.4	11.5	4.7	4.3	7.8
WhatsApp (VoIP)	9.9	5.7	21.2	0.9	1.0	1.6	10.4	10.5	12.6

## Data Availability

Dataset available on request from the authors.
